# Novel lipid and ascorbyl stearate biomimetic vancomycin-loaded nanostructured lipid carrier therapy for bacterial infections and sepsis

**DOI:** 10.1039/d6ra01364c

**Published:** 2026-07-02

**Authors:** Vincent O. Nyandoro, Calvin A. Omolo, Eman A. Ismail, Abdelrahman Tageldin, Mohammed A. Gafar, Jasoda Govender, Thirumala Govender

**Affiliations:** a Discipline of Pharmaceutical Sciences, College of Health Sciences, University of KwaZulu-Natal Private Bag X54001 Durban South Africa; b Department of Pharmaceutical Chemistry and Pharmaceutics, School of Pharmacy, Kabarak University Private Bag 20157 Kabarak Kenya; c United States International University-Africa, School of Pharmacy and Health Sciences, Department of Pharmaceutics P. O. Box 14634-00800 Nairobi Kenya; d Department of Pharmaceutics, Faculty of Pharmacy, University of Gezira Wad Medani Sudan; e Department of Pharmaceutics and Pharmaceutical Technology, School of Pharmacy, Kampala International University Ishaka Uganda

## Abstract

The global burden of methicillin-resistant *Staphylococcus aureus* (MRSA)-associated sepsis necessitates innovative therapeutic strategies. In this study, we developed a multifunctional biomimetic nanostructured lipid carrier (NLC) using a synthesized lipid and ascorbyl stearate to simultaneously deliver vancomycin (VCM) and target the reactive oxygen species (ROS) and the A Disintegrin and Metalloproteinase 10 (ADAM10)-alpha-hemolysin (AHL) pathways implicated in sepsis pathogenesis. The NLC-VCM was formulated *via* a nanoprecipitation-ultrasonication method and underwent comprehensive physicochemical characterization. Its biological performance was evaluated through *in vitro* antibacterial, antioxidant, and anti-inflammatory assays, followed by validation in a mice sepsis model. The optimized NLC-VCM exhibited a monodisperse spherical morphology, sustained biphasic drug release over 48 hours, and high biocompatibility. The formulation demonstrated superior antibacterial efficacy, evidenced by a two-fold lower MIC and complete MRSA clearance within 12 hours compared to bare VCM. Flow cytometry confirmed the potent bactericidal activity, with only 34.4% of MRSA cells remaining viable and the majority (61.4%) being non-viable. Furthermore, *in vitro* antioxidant and anti-inflammatory assays confirmed significant ROS scavenging capacity and suppression of LPS-induced IL-1β and TNF-α in RAW264.7 cells. In the mice sepsis model, NLC-VCM treatment significantly reduced bacterial load (73.9% CFU reduction) and IL-18 levels compared to bare VCM. These findings show the potential of NLC-VCM as a therapeutic platform that synergistically combines antibacterial action with host-directed immunomodulation to combat MRSA infections and sepsis.

## Abstract 2

Dual-targeting nanostructured lipid nanocarrier potentiates VCM efficacy and suppresses MRSA burden, oxidative stress and hyperinflammation in sepsis.

## Graphical abstract

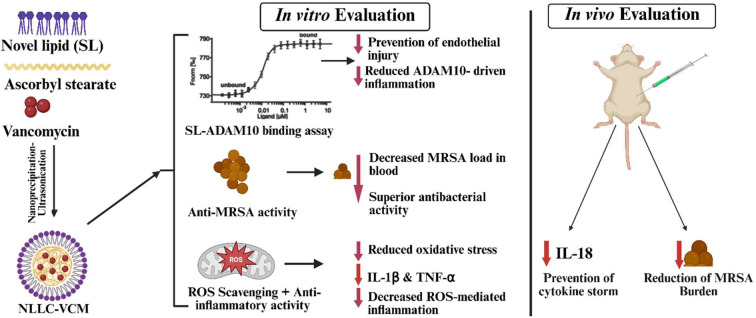

## 1 Introduction

The global burden of bacterial infections is high, a trend closely associated with the increasing prevalence of antimicrobial resistance (AMR).^1^ The emergence of drug-resistant bacterial strains, *e.g.*, MRSA, presents significant challenges, with MRSA being classed as a serious threat requiring urgent intervention.^2^ MRSA remains a critical global health concern, retaining its high-priority status on the WHO 2024 Bacterial Priority Pathogens List.^3^ The Global Burden of Disease (GBD) report identifies MRSA as a top cause of microbial infections globally.^4^ Further, the WHO Global Antimicrobial Resistance and Use Surveillance System Report 2022 reveals that among 76 countries, the median prevalence of MRSA bloodstream infections was 35%.^5^ These infections are linked to higher incidences of sepsis, prolonged hospitalization and increased mortality. The GBD report highlights sepsis as a major cause of morbimortality, with current estimates indicating 166 million cases of sepsis in 2021, leading to about 21.4 million deaths (about 31.5% of the global mortality). The report also highlights a reversal of earlier downward trends, showing a marked rise in both incidence and mortality between 2020 and 2021,with infections leading to sepsis responsible for 15.5 million deaths.^6^ These alarming figures underscore the need for novel therapeutic strategies to address the growing burden of MRSA-associated sepsis.

The current clinical management of MRSA sepsis focuses on bacteria elimination using antibiotics and restoration of immune homeostasis by use of anti-inflammatory drugs, vasoactive agents, and immunomodulators.^7^ Despite these interventions, most sepsis patients exhibit persistent infections and immunosuppression, often progressing to systemic immune dysregulation.^8^ The existing limitations in conventional antibiotic formulations such as subtherapeutic drug concentrations at target sites, inadequate uptake and retention by bacteria, contribute to suboptimal treatment outcomes and exacerbate the development of AMR.^9^ These challenges highlight the need for innovative therapies to simultaneously augment the effectiveness of the existing antibiotics and target the sepsis pathophysiology to enhance therapeutic outcomes.

Nanosized drug delivery systems (NDDS) have been studied for their potential to improve the delivery and efficacy of antibiotics while addressing AMR, owing to their site-specific targeting and controlled release that sustain therapeutic concentrations over prolonged periods.^10^ Recent advancements have focused on biomimetic strategies to optimize the biocompatibility and targeting of the nanocarriers.^11^ Biomimetic systems mimic natural biological processes and structures in bacterial infections.^12^ The biomimetic drug delivery system can be engineered to disrupt the bacterial component signaling, inflammasome assembly, or subsequent signaling cascades, thereby inhibiting pro-inflammatory cytokine secretion.^13,14^ For instance, our research group has designed and evaluated biomimetic NDDS targeting toll like receptors (TLRs) as potential therapy in bacterial infections and sepsis.^15–19^ Thus, utilizing biomaterials with biological properties for the design of nanocarriers offers a promising approach to improve the therapeutic efficacy of the loaded drugs. For example, some antioxidant biomaterials have the ability to modulate cellular and molecular processes in disease states^18^ and therefore when used to construct antibiotic-loaded NDDS, they simultaneously deliver the antibiotic, modulate inflammation and reduce oxidative stress. The optimization of these biomimetic NDDS requires the understanding of the host–pathogen interactions and the associated disease pathophysiology.

The complex pathophysiology of sepsis involves dysregulated host responses to infection, initiated by pattern recognition receptors (PRRs) detecting pathogens.^20,21^ One of the mechanisms is the MRSA alpha-hemolysin (AHL) toxin binding to the A Disintegrin and Metalloproteinase 10 (ADAM10) receptor, which triggers proteolytic cleavage, proinflammatory cytokine release, and endothelial injury.^22,23^ This makes ADAM10 inhibitors of therapeutic interest.^24^ Sepsis is also characterized by enhanced Reactive Oxygen Species (ROS),^25^ which act as secondary messengers to modulate inflammation through pathways like NLRP3 and NF-κB.^26^ The ROS-driven NLRP3 inflammasome activation releases pro-inflammatory cytokines (IL-1β, IL-18) which further amplify oxidative stress and perpetuate the inflammatory cascade. Therapeutically targeting the ROS with antioxidants has shown promise.^27^ Thus, the ADAM10-AHL and ROS-mediated pathways are potential targets for a biomimetic NDDS strategy. Therefore, designing novel materials to target these pathways is an innovative approach to advance this strategy.

In this regard, a novel lipid (SL) targeting the ADAM10-AHL and ROS-NLRP3 pathways, was synthesized and combined with ascorbyl stearate (AS) to construct a biomimetic nanocarrier to deliver an antibiotic and modulate inflammation and reduce oxidative stress in sepsis. SL contains thioether moieties within its architecture that are capable of neutralizing ROS in sepsis.^28^ The AS antioxidant is biocompatible for use in drug delivery systems.^18^ Ascorbate has been documented to scavenge mitochondrial ROS, thereby inhibiting the activation of the NLRP3 inflammasome.^29^ Based on aforementioned mechanistic properties, we hypothesized that combining SL and AS could synergistically enhance ROS scavenging, reduce oxidative stress, restore redox balance, and suppress ROS-associated NLRP3 inflammasome/pyroptosis signaling. Furthermore, given the role of ADAM10 in AHL-mediated pathogenicity, SL was investigated for its potential to modulate the ADAM10 pathway as a strategy to regulate downstream proinflammatory cytokine release. Accordingly, AS and SL were selected to fabricate a nanostructured lipid carrier (NLC) as a multifunctional therapeutic platform.

A NLC consists of solid and liquid lipids that create structurally disordered matrices that optimize drug loading and minimize leakage for the effective delivery of drugs.^30^ Leveraging these advantages, conventional and stimuli-responsive NLC systems have been developed for applications in cancer and infectious diseases.^31–33^ Recently, our group developed a novel biomimetic hybrid NLC based on hyaluronic acid–lysine conjugate, oleylamine and tocopherol succinate for delivery of vancomycin (VCM) against sepsis.^19^ However, SL/AS-based NLC systems remain unexplored as biomimetic carriers for any therapeutic application. In addition, no studies have reported a dual-pathway-targeting biomimetic NLC for drug delivery. Addressing this gap may therefore advance the development of next-generation nanostructured lipid carriers for improved sepsis therapy.

Herein, we present a multifunctional, biomimetic NLC based on the SL and AS to potentiate the efficacy of VCM against MRSA infection and to reduce excessive inflammation and oxidative stress in sepsis. We hypothesize that the AS component will function as a potent ROS scavenger, directly mitigating oxidative stress and its associated inflammatory signaling. Concurrently, the novel SL is engineered to inhibit ADAM10 activity and also scavenge ROS. Through these combined actions, the NLC aims to suppress NLRP3 associated inflammation. This study outlines the synthesis of SL and evaluation of its structure, biocompatibility and biomimicry, followed by the development, optimization, and characterization of NLC-VCM. The nanosystem was evaluated for its biocompatibility, drug release profile, antibacterial activity, and its antioxidant and anti-inflammatory activities. By integrating antibacterial, antioxidant, and anti-inflammatory activities into a single biomimetic nanoplatform, NLC-VCM represents a promising multifunctional therapeutic strategy with dual-pathway targeting capability suitable in conditions with multiple pathological pathways like sepsis and cancer.

## 2 Materials and methods

### 2.1 Materials

All chemicals and solvents utilized were of analytical grade. Ascorbyl stearate (AS), crystal violet, dimethyl sulfoxide, MTT reagent, and lipopolysaccharide from *E. coli* O55:B5, were sourced from Sigma-Aldrich (USA). Cell culture reagents were obtained from Whitehead Scientific (South Africa), while all cell lines were acquired from Highveld Biologicals (South Africa). ADAM10, AHL, IL-1 beta (ELK1270), TNF-alpha (ELK1190), and the LEGENDplex™ MU M1 Panel, were procured from Biocom South Africa. Mitosox Red mitochondrial superoxide was purchased from Thermo Fisher Scientific (USA), and microbiological media, including Mueller-Hinton broth, Mueller-Hinton agar, and Nutrient Broth, were supplied by Oxoid (Ireland). Consumables for microscale thermophoresis (MST) were provided by Nano Temper Technologies (Munich, Germany), and purified water was generated using a Milli-Q water system (Millipore Corp., USA).

### 2.2 Methods

#### 2.2.1 Synthesis and characterization of novel lipid (SL)

**Scheme 1:**
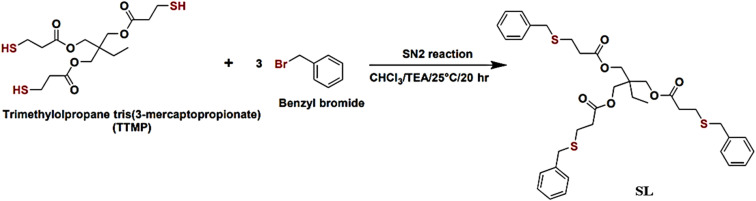
Synthesis of novel lipid, SL. (CHCl_3_: chloroform; TEA: triethylamine).

SL was synthesized *via* a method adopted from ref. 34 as shown in Scheme 1. In brief, a solution containing 3.04 mmol of trimethylolpropane-tris-(3-mercaptopropionate) in 50 mL of chloroform was prepared, followed by the addition of 10.9 mmol of triethylamine. The reaction mixture was stirred for one hour at room temperature before the dropwise addition of 10.9 mmol of benzyl bromide. After 20 hours of continuous stirring, the reaction was quenched with 50 mL of 10% HCl. The organic layer was isolated, washed three times with 50 mL water, and dried over anhydrous Na_2_SO_4_. Chloroform was removed *via* rotary evaporation, yielding the product as a light-yellow liquid (Fig. S1). The novel lipid was characterized by NMR, FT-IR spectroscopy, cytocompatibility and stored for further use.

#### 2.2.2 *In vitro* ADAM10 binding microscale thermophoresis study (MST)

The biomimetic potential of SL was assessed by evaluating its binding affinity to ADAM10 using a Monolith NT.115 MST instrument (NanoTemper Technologies, Germany). AHL was used as the natural substrate for ADAM10. Recombinant human ADAM10 was fluorescently labeled with RED-NHS dye and tested at 50 nM concentration against AHL as the natural substrate reference.

##### 2.2.2.1 Binding check assay

A binding check assay was performed in accordance with the manufacturer's protocol to examine the binding of SL and ADAM10. A 125 µM SL solution was prepared in MST assay buffer and combined 1 : 1 with 50 nM fluorescently labeled ADAM10, while a control sample contained labeled ADAM10 diluted with buffer alone. Following 5 minute incubation at room temperature, quadruplicate measurements were performed using premium capillaries in the Monolith NT.115 instrument at 25 °C with 60% excitation power and 40% MST power.

##### 2.2.2.2 Binding affinity assay

Each ligand was serially diluted with assay buffer to make sixteen dilutions. The final concentrations after mixing 10 µL of each ligand dilution with 10 µL of labeled ADAM10, ranged from 125 µM to 30.5 nM for SL and from 14.3 µM to 0.436 nM for AHL, with a constant final ADAM10 concentration of 25 nM. After 15 minute incubation, the ligand-protein mixtures were loaded into capillaries, and analysis was performed on a Monolith NT.115 instrument at 25 °C and 40% MST power. Kd values were calculated using MO-Affinity Analysis software (v2.1.3).

#### 2.2.3 Preparation of NLC-VCM

The NLC-VCM was prepared using nanoprecipitation-ultrasonication as previously described in ref. 35. Preliminary screening was conducted to determine the optimal surfactant type, surfactant concentration, lipid concentration, and probe sonication time. Following optimization, the chosen surfactant and VCM were mixed with distilled water, and bath sonicated to form the aqueous phase. AS and SL were dissolved in ethanol to prepare the lipid phase which was then introduced dropwise into the aqueous phase under magnetic stirring. The formulation was stirred overnight for complete ethanol evaporation, then probe-sonicated at 30% amplitude and characterized.

#### 2.2.4 NLC-VCM physicochemical characterization

##### 2.2.4.1 Particle size (PS), zeta-potential (ZP), and polydispersity index (PDI)

PS, ZP, and PDI were evaluated using dynamic light scattering (DLS) with a Zetasizer Nano ZS (Malvern Instruments, UK), as described in ref. 16. Prior to analysis, the NLC formulation was diluted with PBS, pH 7.4, and measurements for each parameter were conducted in triplicate.

##### 2.2.4.2 NLC-VCM encapsulation efficiency

The encapsulation of VCM within NLC-VCM was assessed by measuring the encapsulation efficiency (%EE) *via* the centrifugation-ultrafiltration technique.^17^ For this analysis, 2 mL were transferred into Amicon® filters (Millipore Corp., USA) with a 10 kDa molecular weight cutoff. The samples were centrifuged at 3000 rpm for 30 minutes at ambient temperature. The filtrate, containing free VCM, was then analyzed using reversed-phase high-performance liquid chromatography (RP-HPLC) on a Shimadzu HPLC system (Kyoto, Japan) fitted with a photodiode array (PDA) detector. The HPLC analysis was performed using an isocratic mobile phase comprising 0.1% v/v aqueous trifluoroacetic acid and acetonitrile in 85 : 15 ratio, with detection at wavelength of 280 nm and 100 µL injection volume. Isocratic elution was maintained at a flow rate of 1 mL min^−1^. Quantification was performed using a pre-validated linear calibration curve (*y* = 16 877*x* − 41 016; *R*^2^ = 0.9998). All analyses were carried out in triplicate. The encapsulation efficiency (EE%) was calculated using the following equation:
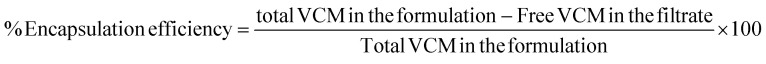


##### 2.2.4.3 NLC-VCM morphology

The morphology of the NLC-VCM was characterized by high-resolution transmission electron microscopy (HRTEM) using a JEOL 2100 instrument operated at 200 kV under cryogenic conditions. For analysis, a diluted suspension was applied to a 300-mesh carbon-coated grid, negatively stained with 2% uranyl acetate, and air-dried prior to imaging.^17^

##### 2.2.4.4 Thermal profile

Differential scanning calorimetry (DSC) analysis was performed on a Shimadzu-DSC-60 instrument to confirm VCM encapsulation within the NLC-VCM. Thermograms for VCM, AS, and lyophilized NLC-VCM were recorded from 25 to 300 °C at a heating rate of 10 °C min^−1^ under a nitrogen purge (20 mL min^−1^).^16^

#### 2.2.5 NLC-VCM biocompatibility

##### 2.2.5.1 *In vitro* cytocompatibility

The cytocompatibility of NLC-VCM was assessed in HEK 293 and HepG2 cell lines using an MTT assay according to a reported protocol.^15^ Cells were maintained in DMEM supplemented with 10% FBS, 1% l-glutamine, 1% penicillin-streptomycin, and 2.5% PBS at 37 °C and 5% CO_2_. At 70–80% confluency, cells were seeded in 96-well plates. After 24 hours, they were treated with NLC-VCM (20–100 µg mL^−1^) for 24 hours. Following treatment, 20 µL of MTT solution (5 mg mL^−1^) was added to each well and incubated for 4 hours. The formed formazan crystals were then dissolved in 100 µL DMSO, and the absorbance was measured at 570 nm after 1 hour of gentle shaking. Untreated cells served as the positive control. Cell viability was calculated as follows:
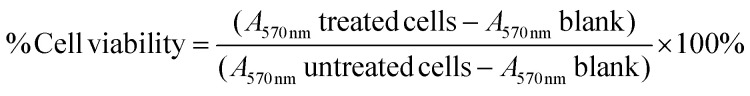


##### 2.2.5.2 *In vitro* hemocompatibility study

The hemocompatibility of NLC-VCM was evaluated using a sheep red blood cell (RBC) assay according to a validated protocol.^16^ RBCs were isolated by centrifuging fresh blood at 2000 rpm for 10 minutes and washing with PBS (pH 7.4). Then, 200 µL of RBCs were mixed with 1.8 mL of NLC-VCM at concentrations ranging from 0.05 to 0.5 mg mL^−1^ in PBS and incubated at 37 °C for 1 hour. After incubation, the samples were centrifuged at 3000 rpm and 4 °C for 5 minutes. The absorbance of the supernatant was measured at 540 nm using a microplate reader (SpectraMax M2, USA), with PBS as negative control and distilled water as positive controls. All experiments were performed in triplicate, and the percent hemolysis was obtained as follows:
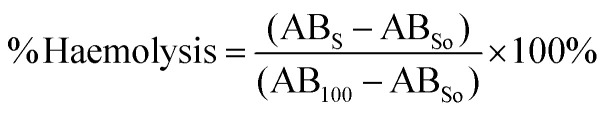
Where AB_s_ is the sample absorbance, ABs_0_ is the negative control absorbance, and AB_100_ is the positive control absorbance.

#### 2.2.6 *In vitro* evaluation of drug release profile and modeling

The release of VCM from NLC was assessed by a dialysis membrane diffusion method.^16^ A 2 mL aliquot of NLC-VCM was dialyzed against 40 mL of PBS (pH 7.4) in a shaking incubator maintained at 37 °C and 100 rpm. Aliquots (2 mL) were withdrawn at predetermined intervals and replaced with fresh PBS. The VCM concentration was quantified using a HPLC, and the release data were fitted to five kinetic models (first-order, Higuchi, Korsmeyer-Peppas, Baker-Lonsdale, and Weibull) using DDsolver software.^36^ The best-fit model was selected based on the highest correlation coefficient (R^2^) and the lowest root mean square error (RMSE).

#### 2.2.7 NLC-VCM physical stability

The stability of the NLC-VCM was investigated for a three-month period at two storage conditions (4 °C and room temperature). Samples were evaluated in triplicate at 0, 4, 8, and 12 weeks for visual appearance, PS, PDI, and ZP.

#### 2.2.8 *In vitro* evaluation of antibacterial activity

##### 2.2.8.1 MIC

Bare VCM, blank NLC, and NLC-VCM activity against methicillin-sensitive *Staphylococcus aureus* (MSSA) and MRSA was determined by broth microdilution and recorded as MIC.^15^ Bacterial suspensions were standardized to 5 × 10^5^ CFU mL^−1^ in Mueller-Hinton Broth, and serial dilutions of each sample were prepared in 96-well plates. Following inoculation and incubation at 37 °C with agitation (100 rpm), 5 µL aliquots from each well were spotted onto Mueller-Hinton Agar at 24 hour intervals for 72 hours. The MIC was defined as the lowest concentration that resulted in no visible bacterial growth after 24 hours of subculture. Tests were done in triplicate.

##### 2.2.8.2 Bacteria kill-time assay

The bactericidal kinetics of NLC-VCM against MRSA were assessed through a time-kill assay using a colony-counting method.^15^ An MRSA suspension (5 × 10^5^ CFU mL^−1^) was treated with bare VCM or NLC-VCM at 5× MIC, using PBS as a negative control. After incubation at 37 °C with shaking (100 rpm) for 24 hours, 0.2 mL aliquots were collected at specified time points, serially diluted, and 0.1 mL plated on MHA. Following a 24 hour incubation, bacterial colonies were enumerated and expressed as log_10_ CFU mL^−1^. All assays were conducted in triplicate.

##### 2.2.8.3 Live/dead fluorescence-activated cell sorting (FACS) assay

Viable MRSA cell counts after 18 hour treatment with VCM and NLC-VCM were quantified by flow cytometry as reported in ref. 17. A bacterial suspension (5 × 10^5^ CFU mL^−1^) was dispensed in 100 µL aliquots into a 96-well plate, followed by addition of 100 µL of either bare VCM or NLC-VCM and incubation at 37 °C under agitation (100 rpm). Aliquots (50 µL) of each treatment at their respective MICs were then transferred to flow cytometry tubes containing 2.75 µL annexin V and 5 µL propidium iodide (PI). Following vortexing and a 15 minute incubation period, 350 µL of sheath fluid was added before analysis on a DxFLEX Flow Cytometer (Beckman Coulter, USA). Untreated MRSA served as the negative control, while heat-shocked MRSA as the positive control. The assay was conducted in triplicate and data analyzed *via* FlowJo_v10.10.0 software.

#### 2.2.9 *In vitro* antioxidant activity

##### 2.2.9.1 DPPH scavenging potential of NLC-VCM

The radical scavenging capacity of NLC-VCM, SL, and AS was assessed *via* a DPPH assay.^16^ Ascorbic acid was the positive control while methanolic DPPH was the negative controls. A 180 µmol L^−1^ DPPH solution was combined with test samples in a 1 : 3 ratio and incubated for 30 minutes at 25 °C in darkness. The resulting absorbance was then quantified at 517 nm using a SpectraMax M2 microplate reader (USA). Radical scavenging activity was calculated as follows:
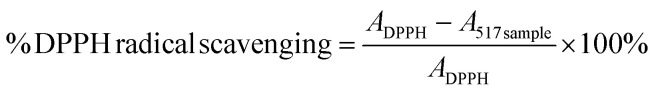


##### 2.2.9.2 *In vitro* assessment of ROS scavenging

The intracellular and mitochondrial ROS scavenging capacity of NLC-VCM was evaluated in RAW 264.7 cells using established protocols^37,38^ with modifications. Cells were seeded on coverslips in 6-well plates (7 × 10^5^ cells per well) and incubated for 24 hours. Subsequently, cells were stimulated with LPS (1 µg mL^−1^) for 4 hours, followed by treatment with NLC-VCM (80 µg mL^−1^) for 16 hours. After 20 hours total, cells were incubated with DCFH-DA (10 µM) and MitoSOX™ Red (2.5 µM) for 20 minutes at 37 °C in the dark. Following three PBS washes, DCFH-DA fluorescence was quantified using a microplate reader, while MitoSOX™ Red fluorescence was visualized by fluorescence microscopy.

#### 2.2.10 Evaluation of *in vitro* anti-inflammatory activity

##### 2.2.10.1 Interleukin 1-beta (IL-1β) and TNF-α levels

To evaluate the anti-inflammatory potential of NLC-VCM, its effect on LPS-induced cytokine release was assessed in RAW 264.7 cells according to a reported method.^39^ Cells were seeded in 96-well plates (5 × 10^3^ cells per well) for 24 hours, stimulated with LPS (1 µg mL^−1^) for 4 hours, and then treated with NLC-VCM (80 µg mL^−1^) for 16 hours. Following centrifugation of the culture medium, cytokine levels (IL-1β, TNF-α) in the supernatant were quantified using ELISA kits.

#### 2.2.11 *In vivo* evaluation of NLC-VCM efficacy

##### 2.2.11.1 Sepsis BALB/c mouse model

The *in vivo* efficacy of NLC-VCM was investigated in a murine sepsis model following an approved protocol (AREC/00003417/2021).^17^ Male BALB/c mice (22–26 g) were randomly divided into four groups (*n* = 6): uninfected (PBS-treated), MRSA-infected (PBS-treated), MRSA-infected (bare VCM-treated), and MRSA-infected (NLC-VCM-treated). Sepsis was induced *via* intraperitoneal injection of 200 µL MRSA suspension. After one hour, groups received their respective treatments (400 µL, i.p.). Mice were monitored for 24 hours for signs of distress before blood collection *via* cardiac puncture for CFU enumeration and cytokine analysis.

##### 2.2.11.2 MRSA load in blood quantification

To quantify bacterial burden, collected blood was serially diluted in PBS, plated on Mueller-Hinton Agar, and incubated at 37 °C for 24 hours. The MRSA load was then calculated as follows:
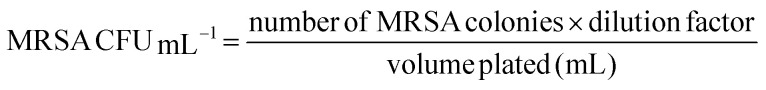


##### 2.2.11.3 IL-18 assay

Blood samples were centrifuged at 10 000 rpm for 5 minutes, and the collected plasma was stored at −80 °C until analysis. Plasma IL-18 levels were quantified using a LEGENDplex™ kit with flow cytometric analysis per manufacturer's instructions.

#### 2.2.12 Statistical analysis

Statistical analysis was conducted using GraphPad Prism (version 8, GraphPad Software Inc., USA). Experiments were done in triplicate (*n* = 3/6). Data are shown as mean ± SD. Normality and variance homogeneity were checked using Shapiro–Wilk and Brown–Forsythe tests. Parametric data were analyzed by one-way ANOVA with Tukey's post-hoc test. Significance was set at *p* < 0.05, with exact *p*-values reported. *In vivo* sample sizes were based on prior studies to ensure statistical power while following 3 Rs and ethical guidelines to minimize animal use.

## 3 Results and discussion

### 3.1 Synthesis and characterization of novel lipid (SL)

The SL was prepared reacting trimethylolpropane tris(3-mercaptopropionate) (TMPTMP) and benzyl bromide *via* a single-step SN_2_ reaction (Scheme 1) and confirmed by ^1^H NMR (Fig. S2c) and FTIR (Fig. S3c). The ^1^H NMR spectrum confirms successful formation of the benzyl thioether derivative of trimethylolpropane tris(3-mercaptopropionate). The spectrum shows aromatic proton multiplets at *δ* 7.2–7.4 ppm and a singlet at *δ* 3.8–4.0 ppm corresponding to the benzylic –CH_2_–S– protons.^40^ Methylene protons adjacent to oxygen appear as a triplet at *δ* 4.1–4.3 ppm,^41^ while those adjacent to sulfur resonate as multiplets at *δ* 2.6–3.0 ppm, consistent with the thioether-linked ester structure. The trimethylolpropane backbone gives characteristic signals at *δ* 1.5–1.7 ppm (central –CH_2_–) and *δ* 0.8–1.0 ppm (terminal –CH_3_). No broad resonance attributable to –SH protons (*δ* 1.5–2.5 ppm)^42^ is observed, indicating complete consumption of thiol groups and confirming successful substitution of benzyl bromide to yield the desired thioether product.

In the FTIR spectrum, the disappearance of the thiol S–H stretching band at 2550–2600 cm^−1^ (refs. 42 and 43) and the C–S–H bending vibration near 801 cm^−1^ indicates complete consumption of the mercapto groups. New absorption bands appear between 602-702 cm^−1^, corresponding to C–S stretching in the thioether linkage, while aromatic C–H stretching at 2965 cm^−1^ and C=C vibrations at 1458 cm^−1^ (ref. 43) confirm the introduction of benzyl groups. The carbonyl stretch at 1727 cm^−1^ and C–O stretching bands in the 1000–1300 cm^−1^ region verify retention of the ester functionalities in the product. Collectively, these spectral features support successful novel lipid synthesis with thioether moieties essential for ROS scavenging.

The cytocompatibility of the SL was evaluated using the MTT assay on RAW264.7 macrophages and HEK293 epithelial cells. The lipid exhibited high cell viability across all tested concentrations in both cell lines (Fig. S4). This confirms that the lipid is well tolerated by diverse cell types, supporting its suitability for biomedical applications such as drug delivery.

### 3.2 *In vitro* evaluation of binding of SL to ADAM10

The ADAM10 receptor, when activated by MRSA α-toxin, promotes endothelial injury and inflammation in sepsis.^22,23,44^ Thus, possible therapies include agents that can bind ADAM10 and competitively inhibit toxin-receptor interaction. To investigate this, the direct binding affinity of the SL for ADAM10 was assessed using MST using the natural substrate AHL as a reference. MST quantifies molecular interactions by measuring ligand-induced changes in thermophoresis, reflected as fluorescence intensity variations (TRIC-Fnorm) across concentration gradient and plotted as a dose–response curve.^45,46^ By determining the binding affinity between SL and ADAM10, the potential of SL to target the ADAM10-AHL signaling axis is predicted.

**Fig. 1:**
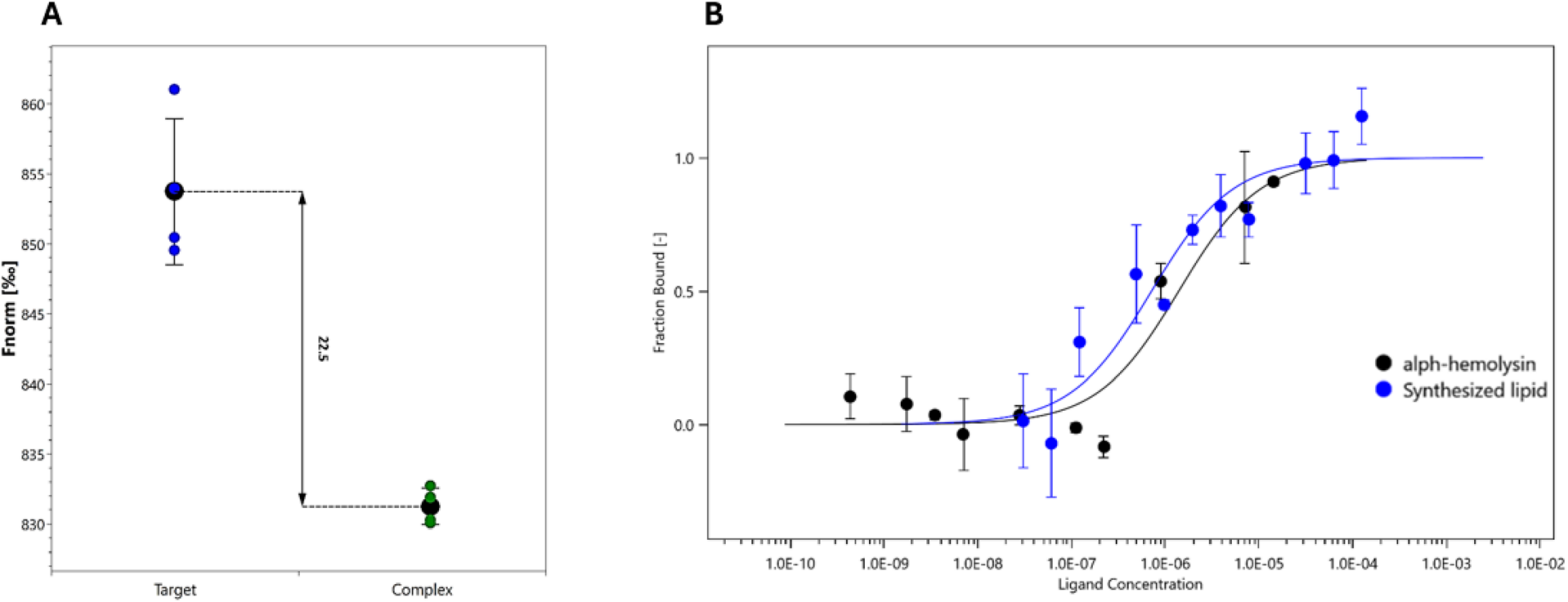
*In vitro* evaluation of binding of SL to ADAM10. (A) MST results of binding check of SL to ADAM10 and (B) relative binding of SL and AHL to ADAM10. Data represent mean ± SD.

To assess the interaction between SL and ADAM10, a binding assay was performed using fixed concentrations of fluorescently labeled ADAM10 and SL. The results, presented in Fig. 1a, confirm successful binding, as indicated by a significant shift in normalized fluorescence (Fnorm) and a binding response of adequate magnitude, indicating a direct affinity of SL for the ADAM10 protein.^44^ This binding affinity was quantitatively evaluated and compared to the natural substrate AHL *via* the Kd analysis. The Kd serves as a quantitative measure of ligand–protein interaction affinity, where a lower value corresponds to stronger affinity.^47^ As illustrated in Fig. 1b, MST analysis demonstrated that the SL binds to ADAM10 with a Kd of 0.70 µM, showing higher affinity than AHL, which exhibited a Kd of 1.38 µM. The stronger interaction of the lipid with ADAM10 suggests its potential to competitively inhibit AHL binding, thereby blocking toxin-mediated activation of ADAM10. This enhanced affinity may result from favorable hydrophobic^48^ or thioether-based interactions within the lipid structure that promote tighter complex formation. Since AHL binding with ADAM10 is a key step in MRSA-induced vascular injury and inflammatory signaling, these results indicate that the SL may mitigate ADAM10-mediated vascular injury and inflammation associated with MRSA α-toxin pathogenesis.

### 3.3 Preparation and optimization of NLC-VCM

The NLC-VCM was prepared using nanoprecipitation-ultrasonication technique. Key parameters including surfactant type, sonication time, lipid concentration, and surfactant concentration that influence nanoparticle stability, homogeneity, and *in vivo* performance^49^ were varied one at a time as shown in Fig. S5 to achieve optimal PS and PDI. Surfactant screening demonstrated that formulations containing Tween-60 produced nanoparticles with a mean size of 93.72 nm and a PDI of 0.515, whereas those prepared with Poloxamer 407 had larger particles of 280.2 nm and a PDI of 0.756. In contrast, the combination of Tween-60 and Span-60 resulted in nanoparticles of 145.8 nm with a PDI of 0.201, indicating enhanced uniformity in particle size distribution attributable to synergistic stabilization from the hydrophilic-lipophilic surfactant blend.

Probe sonication time demonstrated an inverse relationship with both PS and PDI. Extending the duration from 5 to 10 minutes reduced the PS from 231.1 to 158.3 nm and the PDI from 0.286 to 0.277, attributable to intense cavitation forces that promote more efficient nanoparticle breakdown, consistent with prior nanoformulation studies.^50^ In contrast, increasing the lipid concentration from 1 mg mL^−1^ to 3 mg mL^−1^ resulted in a modest PS increase from 158.3 to 169.6 nm, possibly due to higher dispersion viscosity with less effective droplet breakup.^51^ Increasing surfactant concentration produced a non-linear effect on PS and PDI: increasing surfactant from 0.2% w/v to 0.3% w/v reduced both PS and PDI, whereas a further increase to 0.5% w/v led to an increase in PS. This pattern suggests that optimal surfactant levels provide complete nanoparticle coating and stabilization, while excess surfactant may induce micelle formation or depletion flocculation, leading to particle aggregation, as previously documented.^52^ The final optimized formulation achieved a sub-200 nm PS with a narrow size distribution, a critical attribute for enhanced stability and cellular uptake,^53^ underscoring the need for optimization in nanocarrier development.

### 3.4 NLC-VCM demonstrated optimal physicochemical properties

**Fig. 2:**
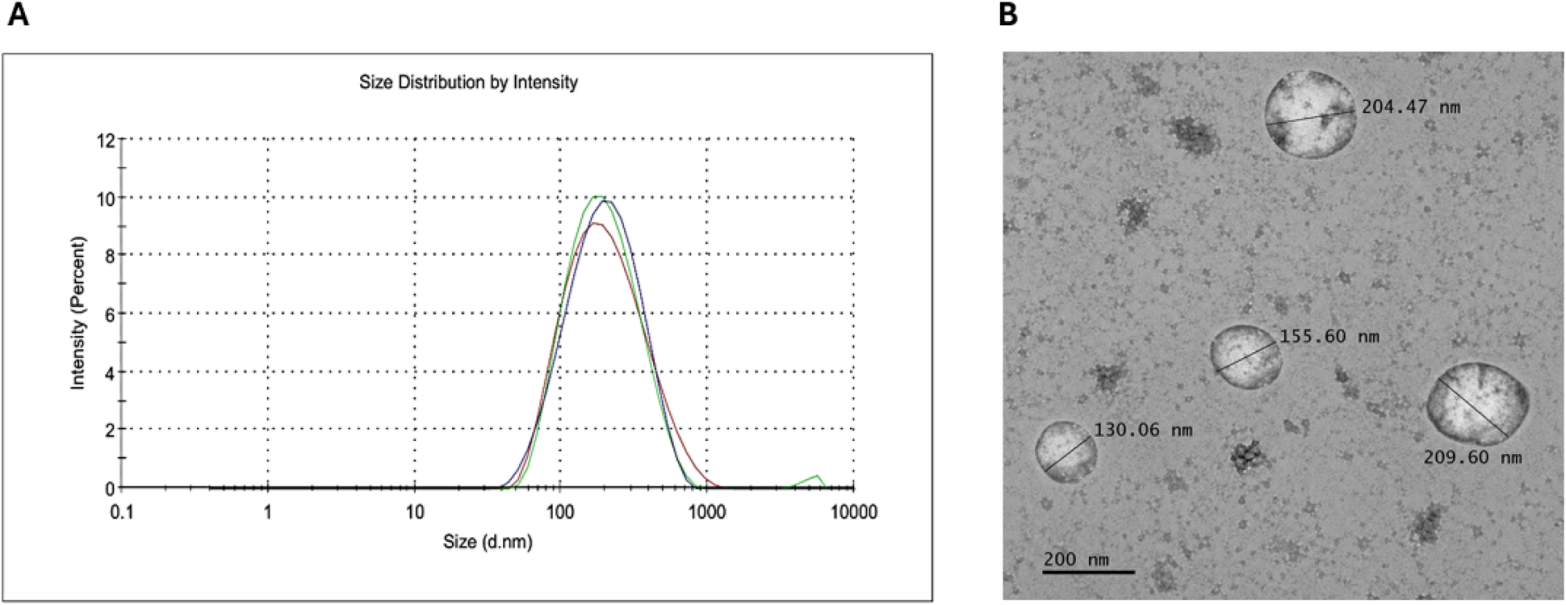
NLC-VCM formulation particle size distribution. (A) Zetasizer analysis showing PS distribution and (B) TEM image showing spherical NLC-VCM with sizes of 130.06 to 209.60 nm.

The physicochemical properties of the optimized NLC-VCM formulation are summarized in Table 1. DLS analysis revealed a mean particle size of 169.1 ± 1.153 nm with a PDI of 0.232 ± 0.013 (Fig. 2a), reflecting a homogeneous size distribution suitable for enhanced cellular uptake and passive targeting *via* the enhanced permeability and retention (EPR) effect.^54^ TEM also revealed that the NLC-VCM nanoparticles possessed a spherical morphology, with directly measured particle sizes ranging from 160.99 to 165.31 nm (Fig. 2b). The relatively small size is attributed to the optimized formulation parameters. The spherical shape and lack of aggregation observed *via* TEM confirmed the efficient stabilizing action of the Tween 60 and Span 60 combination. The zeta potential of −16.6 ± 0.635 mV, suggests moderate colloidal stability, sufficient to prevent aggregation through electrostatic repulsion.^55^ The moderate %EE (55.87%) enhances therapeutic effectiveness with little drug wastage, economical for industrial scale up.

**Table 1:** Optimized NLC-VCM physicochemical characteristics. Data are mean ± SD, *n* = 3

PS (nm)	PDI	ZP (mV)	%EE
169.1 ± 1.153	0.232 ± 0.013	−16.6 ± 0.635	55.87

The successful encapsulation of VCM within the lipid nanocarrier was verified using Differential Scanning Calorimetry (DSC). The thermal profiles of the individual components *i.e.* VCM, AS, and the cryoprotectant trehalose were compared with that of the final NLC-VCM formulation (Fig. S6). VCM exhibited a characteristic broad endothermic event at 87.89 °C, attributed to its flash point,^15^ while AS showed a distinct phase transition at 121.18 °C. The thermogram of trehalose presented broad endothermic peaks, typical for amorphous sugars undergoing water loss. The DSC curve for the NLC-VCM formulation showed the absence of the distinct VCM endothermic peak, suggesting that the drug was incorporated within the lipid matrix.

The physical stability of the NLC-VCM formulation was evaluated over 12 weeks by monitoring its PS, PDI, and ZP under storage at both room temperature and 4 °C (Table 2). Storage at room temperature led to a substantial increase in PS (from 169.0 ± 1.153 nm to 404.1 ± 24.98 nm) and PDI (from 0.232 ± 0.013 to 0.902 ± 0.118), suggesting particle aggregation and growth. Storage at 4 °C effectively preserved the formulation's properties as PS exhibited a slight increase to 201.1 ± 1.852 nm, PDI remained low (0.287 ± 0.011), and ZP maintained around −18 mV. These results, demonstrate stability at 4 °C, align with previous findings on lipid-based nanocarriers,^56^ and confirm the necessity of refrigerated storage for maintaining the integrity of NLC-VCM.

**Table 2:** Physical stability of NLC-VCM

Storage	Room temp			4 °C		
Time	PS	PDI	ZP	PS	PDI	ZP
Day 1	169.0 ± 1.153	0.232 ± 0.013	−16.6 ± 0.635	169.0 ± 1.153	0.232 ± 0.013	−16.6 ± 0.635
4 weeks	316.4 ± 4.243	0.454 ± 0.230	−16.8 ± 0.289	214.7 ± 7.071	0.298 ± 0.020	−17.2 ± 1.580
8 weeks	395.0 ± 32.08	0.705 ± 0.219	−15.1 ± 0.493	213.1 ± 5.771	0.269 ± 0.023	−18.6 ± 0.351
12 weeks	404.1 ± 24.98	0.902 ± 0.118	−13.7 ± 1.050	201.1 ± 1.852	0.287 ± 0.011	−18.9 ± 0.651

### 3.5 NLC-VCM showed adequate preliminary cytocompatibility and hemocompatibility

**Fig. 3:**
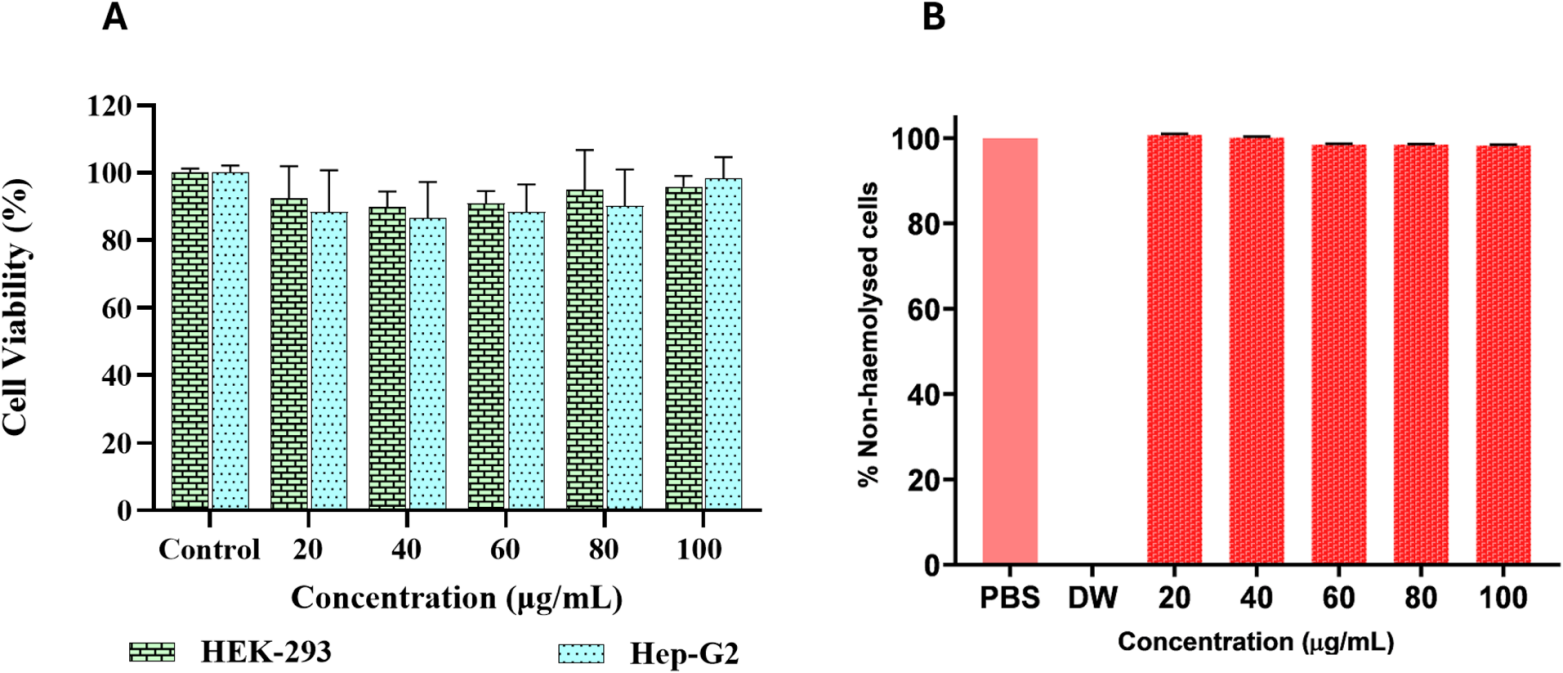
Evaluation of NLC-VCM biocompatibility. (A) MTT assay demonstrating high cell viability (>85%) in HepG2 and HEK293 cells across all concentrations. (B) Hemolysis assay showing minimal RBC lysis (<1%) across all concentrations. Values are mean ± SD (*n* = 3).

The biocompatibility of NLC-VCM, a prerequisite for parenteral applications,^57^ was confirmed through cytocompatibility and hemocompatibility assessments. An MTT assay on HepG2 and HEK293 cells demonstrated a cell viability exceeding 85% at all tested concentrations (Fig. 3a), classifying the formulation as non-cytotoxic (>70% viability).^46^ Also, hemolysis assay revealed negligible hemolysis, with less than 1% RBC lysis (Fig. 3b). These findings are consistent with prior reports that NLC formulations show low cytotoxicity in standard cell-based assays and minimal haemolytic activity in RBC assays.^58,59^ While these findings show biocompatibility, evaluation according to ISO 10993, is needed to fully establish the safety profile of the nanocarrier.

### 3.6 NLC exhibited a sustained biphasic VCM release

**Fig. 4:**
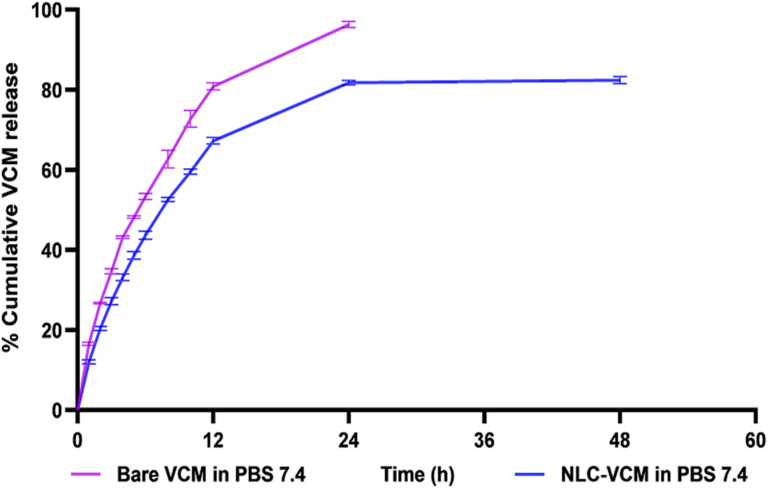
Release of VCM from bare VCM solution and NLC-VCM. Data are mean ± SD, *n* = 3.

The release kinetics of VCM from the NLC was characterized through *in vitro* studies to assess its sustained delivery potential. Using a dialysis method over 48 hours, bare VCM demonstrated a rapid burst release, with 80.83% of the drug released within 12 h and 96.27% within 24 h. In contrast, the NLC-VCM formulation exhibited a sustained, biphasic release pattern, characterized by an initial burst release of 67.24% in the first 12 h, followed by a much slower phase, reaching cumulative releases of 81.67% at 24 h and 82.39% at 48 h (Fig. 4). This biphasic release pattern, which is sustained over time, delivers an initial burst for quickly reaching therapeutic levels, then continues with extended drug release to keep concentrations effective for more than 24 hours. In contrast, bare VCM is fully released within 24 hours, necessitating repeated dosing. Thus, the biphasic pattern improves antibacterial activity and reduces dosing frequency compared to the bare drug. Kinetic analysis indicated that the release data best fit the Weibull model (*R*^2^ = 0.9745, lowest RMSE; Table S1), a mechanism involving both drug diffusion and matrix relaxation of the nanocarrier.^60^ Similar release profiles have been reported in NLC nanosystems.

### 3.7 NLC-VCM had enhanced antibacterial activity against MRSA compared to bare VCM

The MIC of NLC-VCM against MRSA was determined and compared to bare VCM. As summarized in Table 3, the NLC-VCM formulation demonstrated enhanced antibacterial efficacy, exhibiting a 2-fold lower MIC value (7.81 µg mL^−1^) than that of bare VCM (15.63 µg mL^−1^) across 24, 48, and 72 hour time points. This improvement in efficacy is possibly due to the sustained release profile of the nanocarrier, which prolongs the exposure of bacteria to effective VCM concentration. The findings align with existing literature,^17,61^ corroborating that nanoencapsulation can significantly enhance the antibacterial activity of VCM.

**Table 3:** Antibacterial activity of VCM, NLC-VCM, and NLC. Data are mean ± SD

Bacteria strain	MSSA (MIC in µg mL^−1^)			MRSA (MIC in µg mL^−1^)		
Sample|time	24 h	48 h	72 h	24 h	48 h	72 h
VCM	1.95	1.95	1.95	15.63	15.63	15.63
NLC-VCM	1.95	1.95	1.95	7.81	7.81	7.81
Blank NLC	NA	NA	NA	NA	NA	NA

**Fig. 5:**
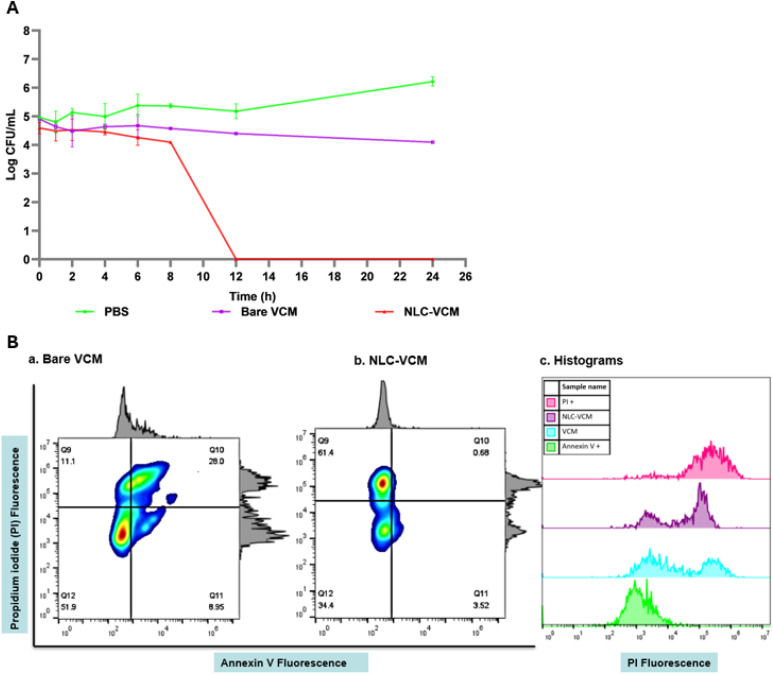
Time-kill kinetics and live/dead of NLC-VCM. (A) MRSA kill over time after treatment with PBS, VCM and NLC-VCM, with complete bacterial eradication within 12 h for the NLC-VCM. (B) Quantitative analysis of MRSA viability by flow cytometry showing low viability in NLC-VCM treated MRSA cells compared to bare VCM.

Moreover, a time-kill assay was conducted to compare the bactericidal kinetics of NLC-VCM with bare VCM against MRSA. The killing profiles, presented as log CFU mL^−1^*versus* time (Fig. 5a), demonstrated that NLC-VCM resulted in complete bacterial eradication within 12 hours. This enhanced bactericidal activity is consistent with the previously observed lower MIC values, confirming the superior efficacy of the nanoformulation. The rapid killing action of NLC-VCM is consistent with prior reports^62^ and holds clinical significance for mitigating the risk of persistent infections. In contrast, bare VCM failed to achieve complete bacterial clearance even after 24 hours. The relatively high MIC of bare VCM against the MRSA strain (15.63 µg mL^−1^) indicates reduced susceptibility, which likely contributed to the limited bactericidal activity observed in the time-kill assay. Accordingly, bare VCM produced only a modest reduction (∼1 log CFU mL^−1^ over 24 h).

Furthermore, flow cytometry with live/dead staining quantitatively evaluated the bactericidal efficacy of NLC-VCM by distinguishing between live and dead cells of MRSA populations.^63^ Treatment with NLC-VCM resulted in fewer live MRSA cells (Fig. 5b(b)) compared to treatment with bare VCM as shown in Fig. 5b(a). These findings align with previous findings on VCM-loaded nanocarriers,^19^ and corroborate the enhanced antibacterial activity of NLC-VCM, reinforcing its therapeutic potential against MRSA infections.

### 3.8 NLC-VCM demonstrated excellent antioxidant activity

**Fig. 6:**
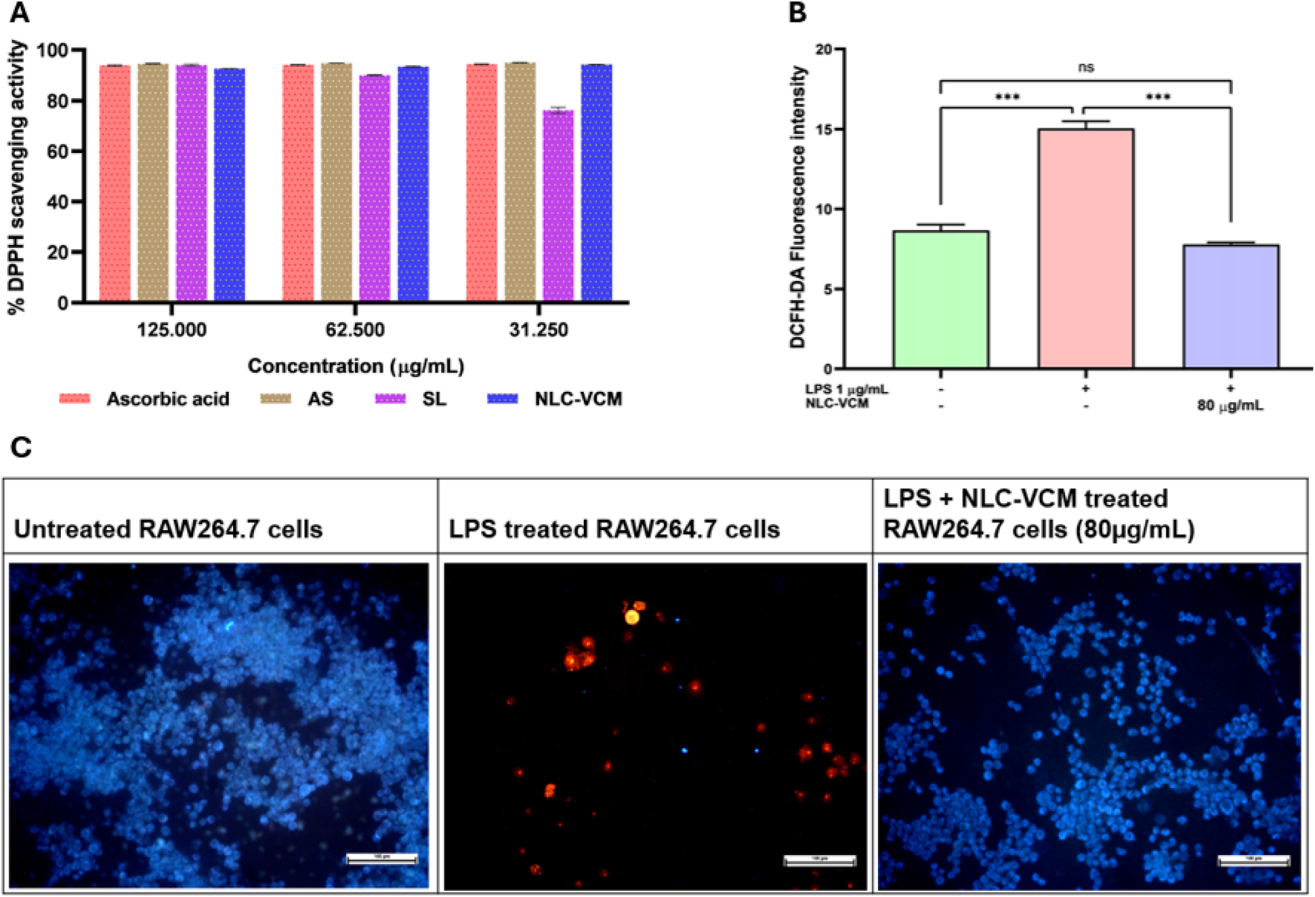
NLC-VCM antioxidant activity. (A) DPPH-radical scavenging activity of NLC-VCM, SL and AS. (B and C) Intracellular and mitochondrial ROS scavenging by NLC-VCM. LPS increased ROS in RAW264.7 cells, shown by high DCFH-DA intensity (B) and red fluorescence (C). NLC-VCM substantially reduced the ROS levels (*p* < 0.001 *vs.* LPS-treated cells).

The antioxidant capacity of NLC-VCM was evaluated using a DPPH radical scavenging assay, given the role of oxidative stress in infection progression and sepsis-related organ damage.^64^ NLC-VCM exhibited radical scavenging activity equivalent to ascorbic acid (Fig. 6a), indicating its potential to ameliorate oxidative stress in MRSA infections. This strong antioxidant was contributed by both AS and SL components of the nanocarrier. AS, a known antioxidant used in food and cosmetic applications,^65^ donates electrons to neutralize and stabilize DPPH radicals,^66^ while the SL contributes thioether moieties that also quench free radicals and scavenge ROS.^28^ These findings are consistent with previous reports demonstrating DPPH neutralization by nanocarriers fabricated from antioxidant biomaterials.^15,67,68^

Moreover, the ROS scavenging capacity of NLC-VCM was investigated to determine its potential cytoprotective role, as excessive ROS contributes to organ damage and activates inflammatory pathways such as the NLRP3 inflammasome in sepsis.^69^ Using RAW264.7 macrophages stimulated with 1 µg mL^−1^ LPS, treatment with 80 µg mL^−1^ of NLC-VCM significantly reduced both intracellular and mitochondrial ROS levels, as measured by DCFH-DA (*p* < 0.001 compared to LPS-treated cells) (Fig. 6b) and MitoSOX™ Red fluorescence intensity (Fig. 6c). The formulation restored redox balance to baseline levels, comparable to untreated cells. This cytoprotective effect is ascribed to the intrinsic antioxidant properties of its SL and AS constituents. This is consistent with prior studies on antioxidant-based nanocarriers,^67,70^ confirming the formulation's potent antioxidant and cytoprotective properties.

### 3.9 NLC-VCM suppressed secretion of pro-inflammatory cytokines in LPS-stimulated cells

**Fig. 7:**
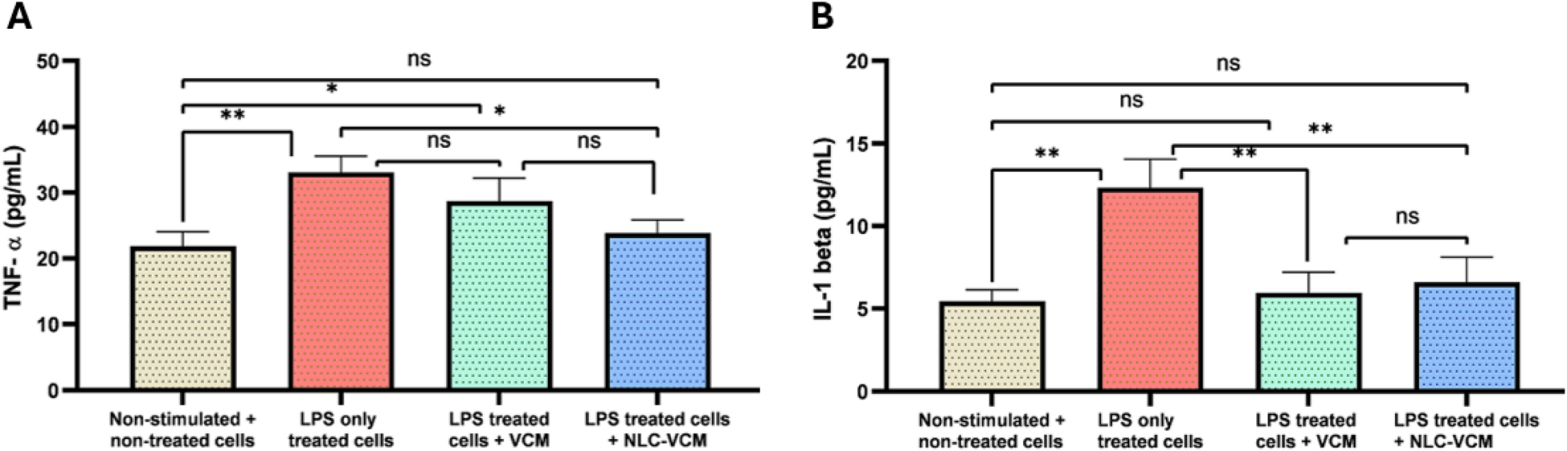
*In vitro* anti-inflammatory activity of bare VCM and NLC-VCM in LPS-stimulated RAW264.7 macrophages. NLC-VCM treatment significantly suppressed TNF-α (*p* < 0.05) and IL-1β (*p* < 0.001) compared to the LPS-stimulated control. Data represent mean ± SD (*n* = 6).

The capacity of NLC-VCM to ameliorate infection-associated inflammation was evaluated in LPS-stimulated RAW 264.7 macrophages by quantifying cytokine production. ELISA analysis confirmed that LPS stimulation significantly elevated IL-1β and TNF-α release compared to untreated controls. While bare VCM did not significantly reduce TNF-α levels (*p* > 0.05), NLC-VCM treatment resulted in a significant suppression of this cytokine (*p* < 0.05; Fig. 7a). Both bare VCM and NLC-VCM demonstrated a potent and significant inhibition of IL-1β (*p* < 0.001; Fig. 7b). The superior anti-inflammatory activity of NLC-VCM is likely mediated through its ROS-scavenging capability, which interrupts ROS-driven activation of inflammatory pathways, thereby reducing downstream IL-1β and TNF-α release. These results corroborate previous findings on the ability of antioxidant nanosystems to modulate ROS-mediated inflammatory cascades.^71,72^

### 3.10 NLC-VCM decreased MRSA burden and inflammation in septic mice

**Fig. 8:**
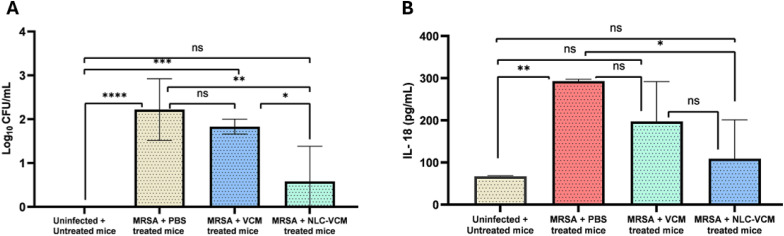
Blood MRSA burden and plasma IL-18 levels in MRSA-infected mice. (A). NLC-VCM reduced bacterial load by 73.9% compared to PBS (*p* < 0.001) and bare VCM (*p* < 0.05), while bare VCM showed nonsignificant effect. (B) NLC-VCM significantly suppressed IL-18 compared to bare VCM (*p* < 0.05). Data represent mean ± SD (*n* = 3).

The *in vivo* antibacterial efficacy of NLC-VCM was assessed in a murine sepsis model by quantifying bacterial load in the blood, with the results shown in Fig. 8a. MRSA-infected mice treated with PBS exhibited a high bacterial burden confirming successful establishment of sublethal infection. Treatment with bare VCM led to only a slight, non-significant reduction in CFUs. In contrast, NLC-VCM treatment significantly suppressed the bacterial burden, achieving a 73.9% reduction in CFUs compared to the PBS group, which was statistically significant against both PBS (*p* < 0.001) and bare VCM (*p* < 0.05). The strong reduction in blood CFUs is attributable to the enhanced systemic therapeutic performance of NLC-VCM. The enhanced antibacterial activity observed for NLC-VCM compared to bare VCM can be attributed to both the mechanism of VCM and the functional properties of the NLC. While bare VCM inhibits MRSA by binding to D-Ala-D-Ala termini and blocking peptidoglycan crosslinking, its efficacy may be limited by suboptimal tissue distribution and reduced retention at the infection site. In contrast, NLC-VCM provides a platform that improves therapeutic performance through targeting the infection site through passive targeting, interaction with bacterial cell surfaces and sustained release of VCM. In addition, the antioxidant and anti-inflammatory properties, contributes to improved outcomes in complex pathological environments such as sepsis, where both microbial burden and host inflammatory responses drive disease progression. This finding is consistent with previous reports on antibiotic-loaded nanocarriers in MRSA infection models.^73,74^

Plasma levels of IL-18, a key mediator of inflammatory pathology in MRSA sepsis,^75^ were measured to evaluate the immunomodulatory effect of NLC-VCM. MRSA infection significantly elevated the cytokine. While bare VCM showed no significant effect, NLC-VCM treatment significantly suppressed IL-18 (*p* < 0.05, Fig. 8b), attributable to its dual antibacterial and ROS-scavenging capacity that synergistically inhibits inflammasome activation. The suppression of IL-18 underscores the potential of NLC-VCM to mitigate sepsis-associated hyperinflammation. This dual therapeutic mechanism of NLC-VCM holds significant clinical relevance in sepsis management. Beyond its direct antibacterial action, the formulation targets the dysregulated host immune response through ROS scavenging, which disrupts the signaling pathway that leads to IL-18 release. By concurrently eliminating pathogens and mitigating the associated cytokine storm, NLC-VCM represents a novel therapeutic strategy that addresses both the infectious trigger and its inflammatory consequences, thereby potentially improving clinical outcomes.

While these findings highlight the therapeutic potential of NLC-VCM, this study is limited by the absence of immunohistochemical data which would have provided insight into tissue-level inflammation or injury and systemic protective effects. Future histopathological studies would further strengthen understanding of the formulation's therapeutic potential.

## 4 Conclusion

This study demonstrates the successful development of a multifunctional biomimetic nanocarrier, NLC-VCM, engineered from biomaterials with intrinsic antioxidant and anti-inflammatory properties. The optimized formulation demonstrated good physical stability and biocompatibility, sustained VCM release, and potent anti-MRSA activity, including rapid bactericidal action validated through time-kill and flow cytometric assays. The NLC-VCM also had a significant ROS scavenging capacity, which correlated with its ability to suppress NLRP3-driven inflammation, as evidenced by reduced IL-1β and TNF-α secretion in RAW264.7 macrophages. In a murine sepsis model, NLC-VCM significantly reduced bacterial burden and mitigated IL-18 release, compared to bare VCM. Taken together, these preliminary results underscore the promise of this multifunctional nanocarrier in enhancing antibiotic treatment by concurrently addressing bacterial elimination as well as the inflammation and oxidative stress linked to sepsis. Subsequent studies will focus on confirming the role of ADAM10 in the observed therapeutic outcomes using ADAM10-targeted assays, such as evaluating its expression, measuring its activity, or analyzing substrate cleavage.

## Author contributions

Vincent O. Nyandoro: conceptualization, data collection, analysis, validation, and manuscript writing. Calvin A. Omolo: conceptualization and supervision, funding acquisition, and manuscript review. Abdelrahman Tageldin: methodology and data collection. Eman A. Ismail: methodology and manuscript review. Mohammed A. Gafar: methodology and collection of data. Jasoda Govender: methodology and data collection. Thirumala Govender: conceptualization, supervision, secured funding, validation of results, and revised the manuscript.

## Conflicts of interest

The authors declare no competing interests that could influence the work reported in this paper.

## Supplementary Material

RA-016-D6RA01364C-s001

## Data Availability

Data for this article including figures and tables are included in the main text submitted. The supporting data has been provided as part of the supplementary information (SI). Supplementary information is available. See DOI: https://doi.org/10.1039/d6ra01364c.
