# Supplementary material for: Novel lipid and ascorbyl stearate biomimetic vancomycin-loaded nanostructured lipid carrier therapy for bacterial infections and sepsis
Source: RSC Adv. 2026 Jul 2;16(34):32815–29. doi: 10.1039/d6ra01364c (PMC13326265; doi:10.1039/d6ra01364c)
Supplement: RA-016-D6RA01364C-s001 [file RA-016-D6RA01364C-s001.pdf]

Supplementary Information (SI) for RSC Advances.
This journal is © The Royal Society of Chemistry 2026

Supplementary Material

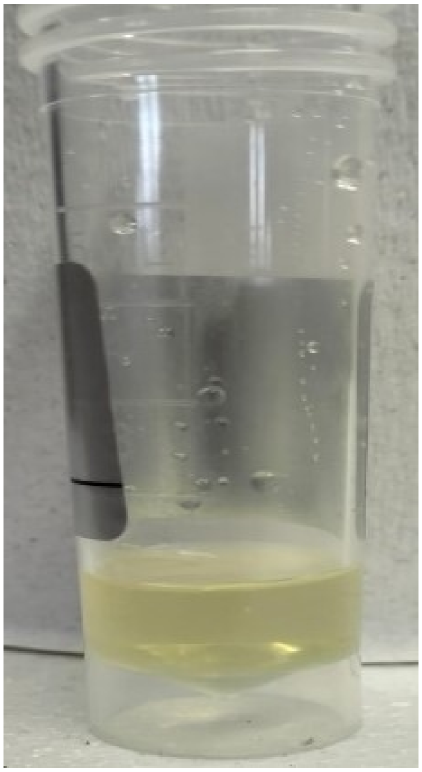

Figure S1. Photograph of the synthesized light-yellow lipid (SL).

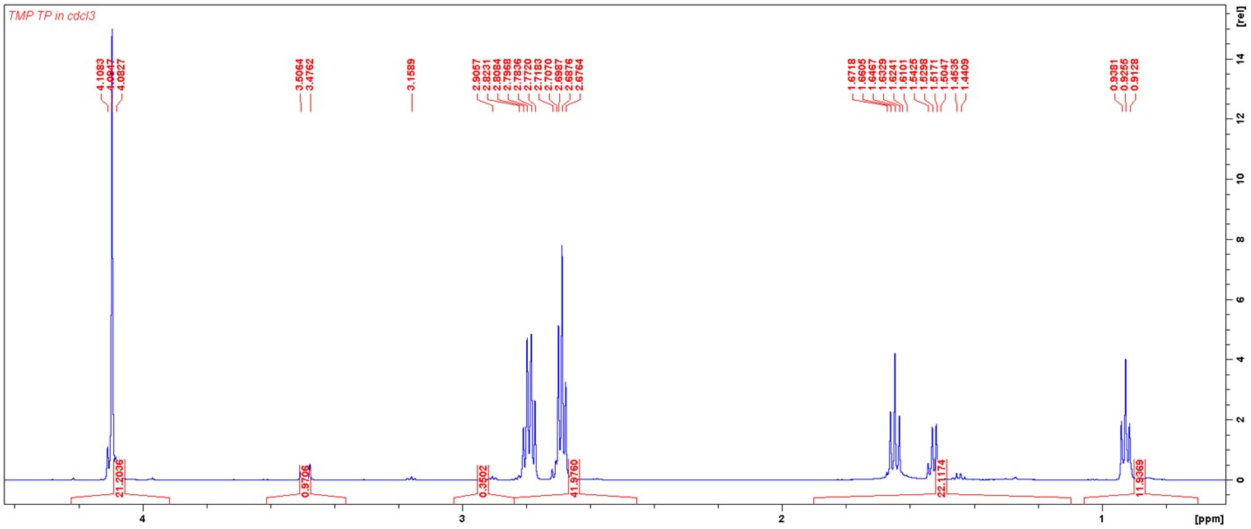

Figure S2a. ^{1}H NMR spectrum of Trimethylolpropane tris(3-mercaptopropionate).

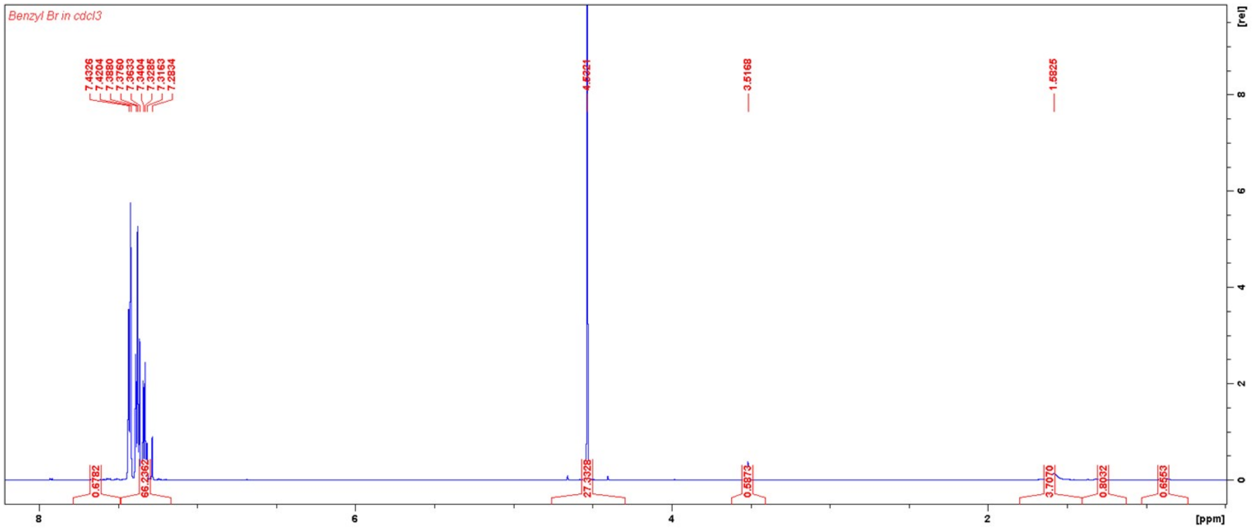

Figure S2b. ^{1}H NMR spectrum of benzyl bromide.

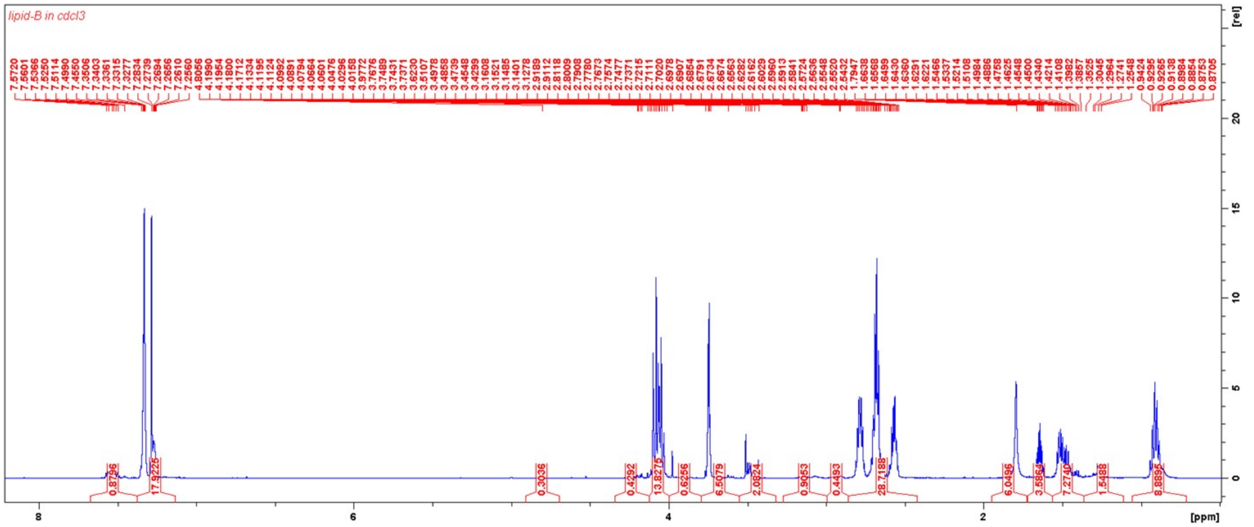

Figure S2c. ^{1}H NMR spectrum of the synthesized lipid (SL) in $CDCl_3$ showing characteristic proton signals.

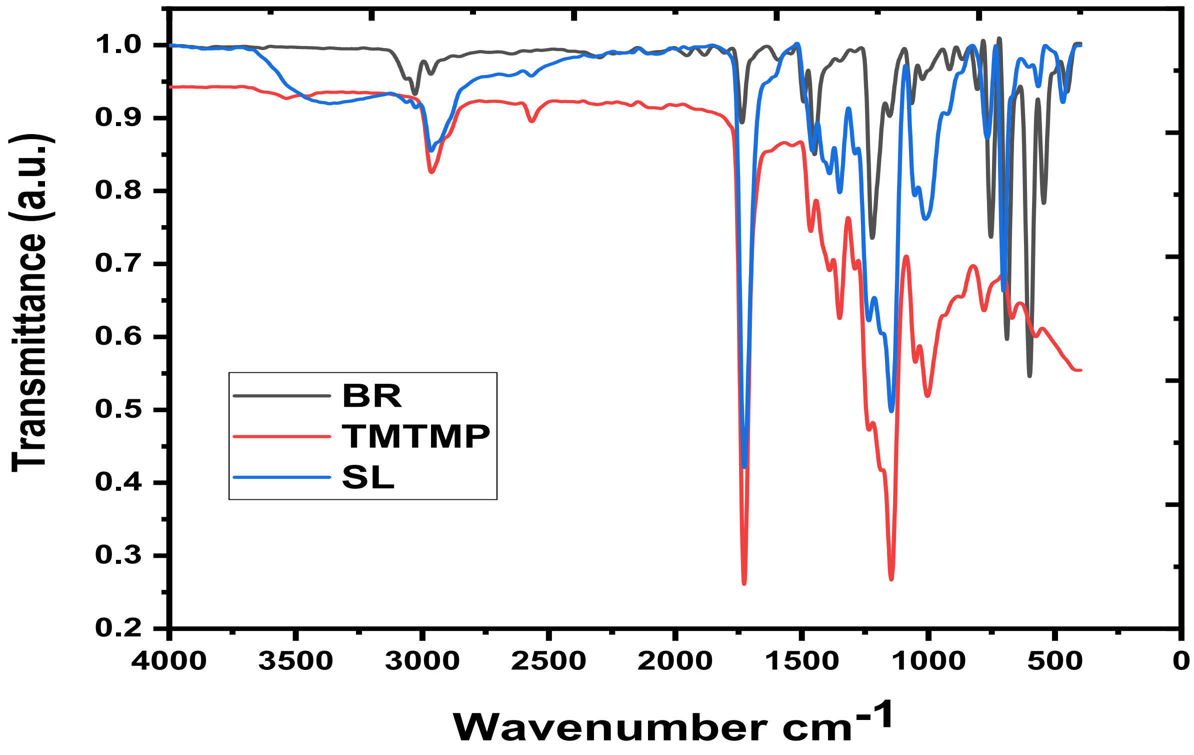

Figure S3. Consolidated FTIR spectra of benzyl bromide (BR), trimethylolpropane tris(3-mercaptopropionate) (TMTMP), and the synthesized lipid (SL), highlighting the characteristic absorption bands of the starting materials and the synthesized product.

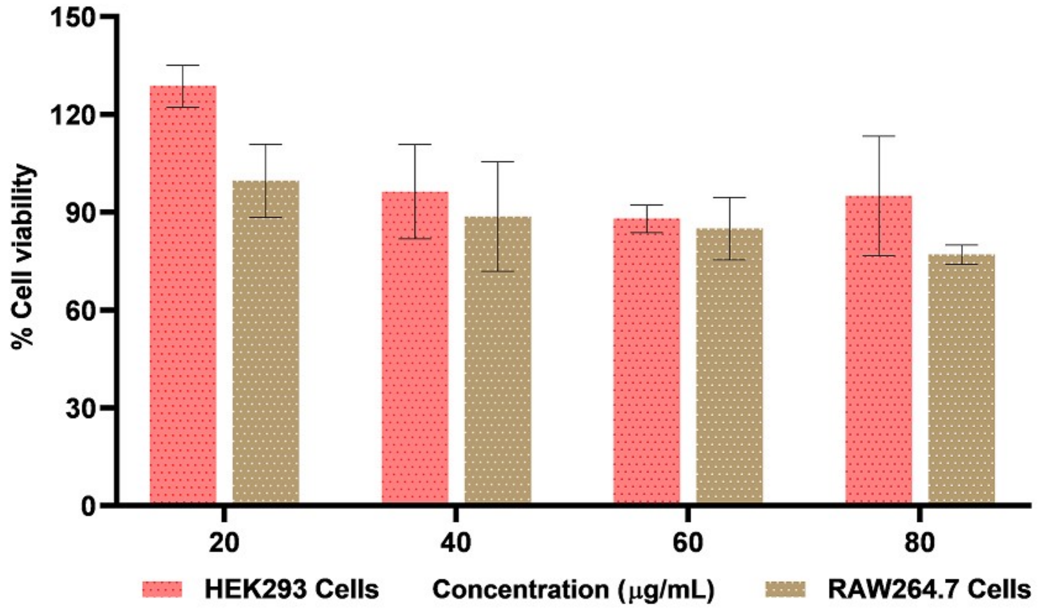

Figure S4. Cytocompatibility of SL on HEK293 and RAW264.7 cells at different concentrations.

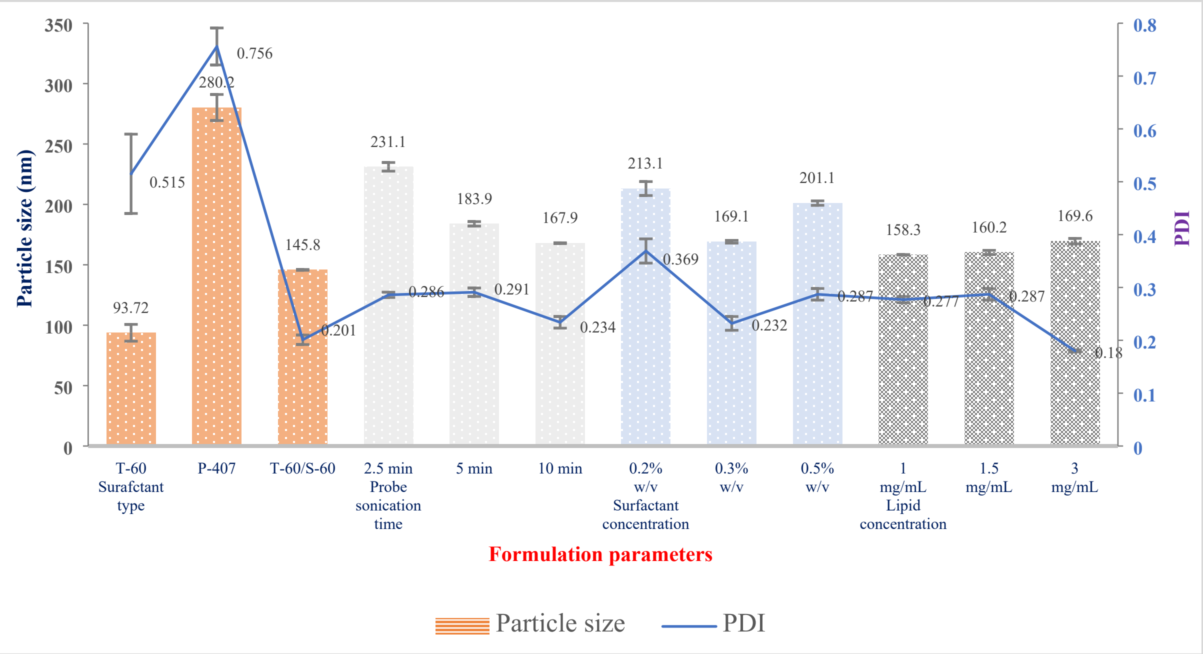

Figure S5. NLC-VCM formulation optimization by evaluating the effects of surfactant type, probe sonication time, surfactant concentration, and lipid concentration on PS and PDI. Data are presented as mean ± SD (n = 3).

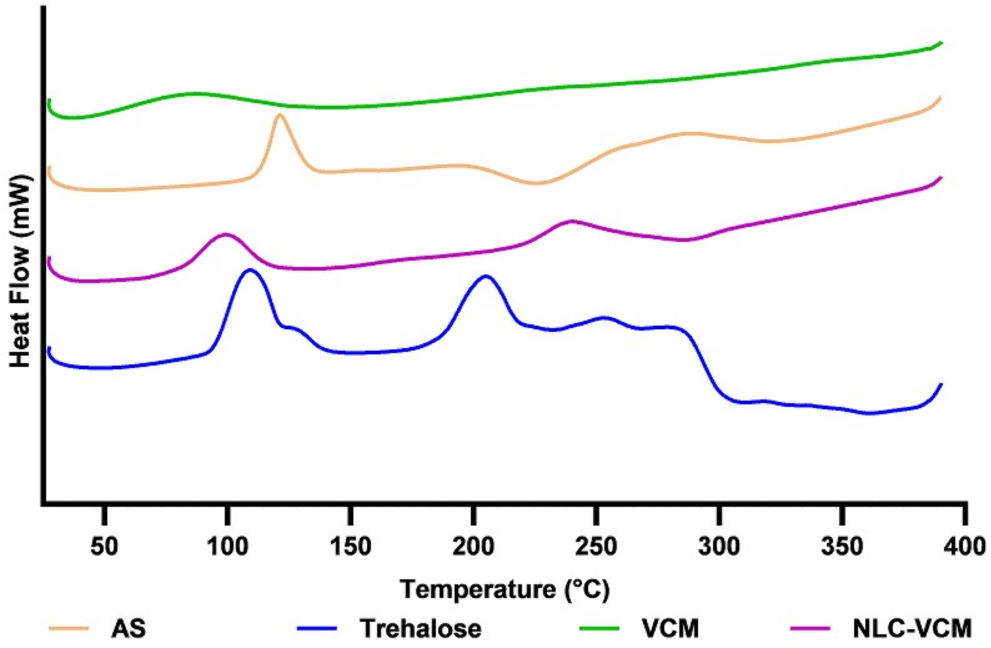

Figure S6. DSC thermograms of AS, trehalose, VCM and NLC-VCM, confirming the entrapment of VCM into the nanocarrier

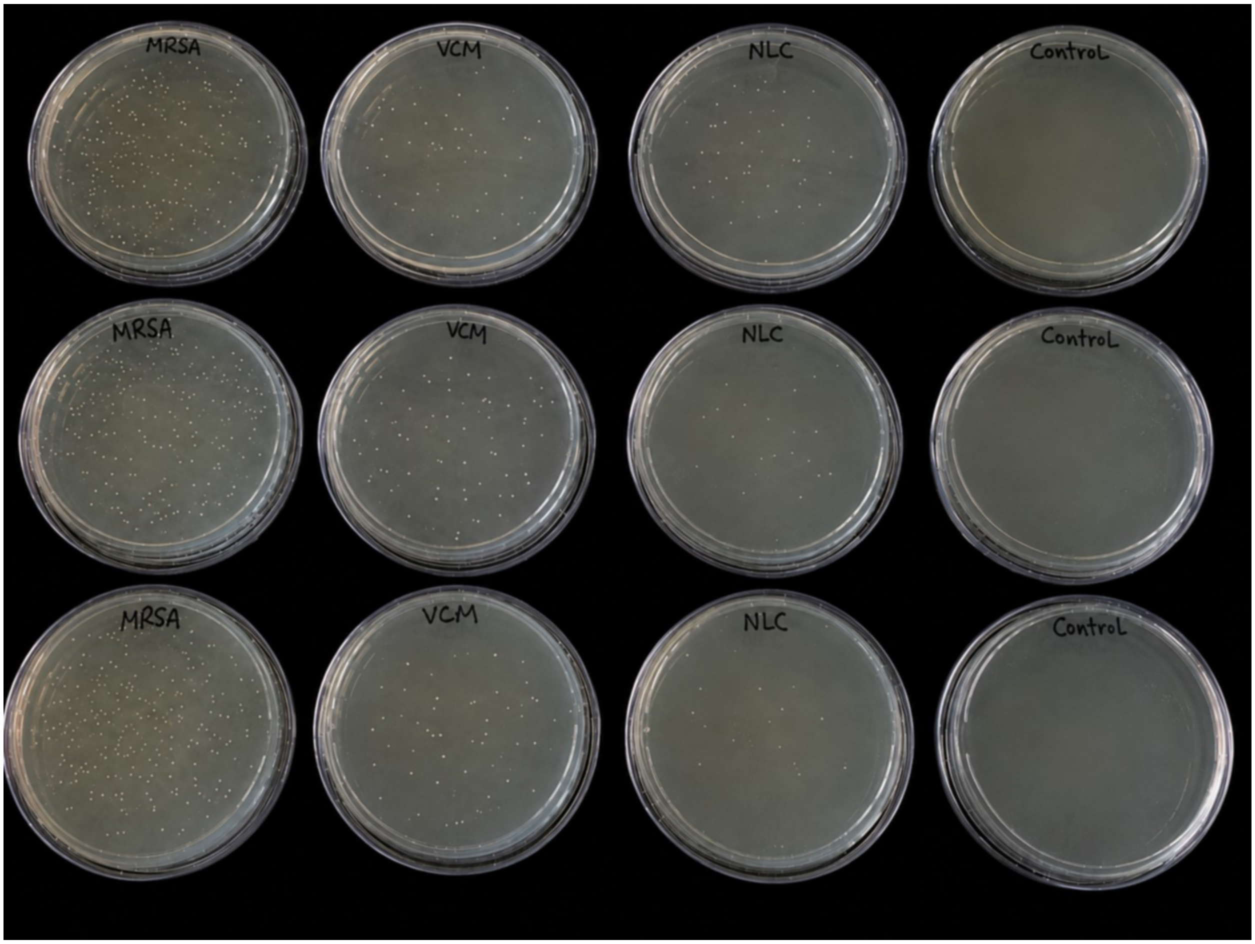

Figure S7. Representative images of mice blood MRSA colonies on culture plates across different treatment groups 24 h post infection + treatment. MRSA plates exhibit the highest colony density, followed by VCM and then NLC, indicating extent of antibacterial activity. Control plates show no visible colony growth, confirming sterility and validity of the assay conditions.

Table S1. VCM release kinetics, the release best fits the Weibull model

**Drug release kinetic model**	**R-squared (R^2)**	**Root mean square error (RMSE)**
First order	0.9355	6.0409
Higuchi	0.8064	10.4650
Korsmeyer-Peppas	0.8862	8.4580
Baker-Lonsdale	0.9301	6.2880
Weibull	0.9745	4.2431
